# Successful removal of a migrated stent retriever tip using wire-guided forceps

**DOI:** 10.1055/a-2418-0807

**Published:** 2024-10-08

**Authors:** Tomohiro Ishii, Kazuya Sugimori, Arisa Omata, Shoichiro Yonei, Takashi Kurosawa, Shin Maeda

**Affiliations:** 1 89460 Department of Gastroenterology, Saiseikai Yokohamashi Nanbu Hospital, Yokohama, Japan; 2 Department of Gastroenterology, Yokohama City University Graduate School of Medicine, Yokohama, Japan

Stent retriever dilation (Soehendra stent retriever, SSR-7; Cook, Tokyo, Japan) has demonstrated effectiveness in treating main pancreatic duct stenosis in chronic pancreatitis 1 2 . However, there have been few reports on stent retriever tip breakage 3 . In this study, we describe a case of dislodgment of the stent retriever tip during main pancreatic duct stenosis owing to chronic pancreatitis, including the removal method.

A 56-year-old man was referred to our hospital for obstructive pancreatitis because of chronic pancreatitis-associated pancreatic stones. Initial endoscopic retrograde cholangiopancreatography (ERCP) demonstrated stenosis of the main pancreatic duct in the pancreatic body. A 5-Fr pancreatic stent was placed in the pancreatic head because it failed to pass through the body stenosis. Subsequently, contrast-enhanced abdominal computed tomography (CT) demonstrated persistent inflammation around the pancreatic body and tail ( Fig. 1 ). Another ERCP was performed to place a pancreatic stent for body stenosis. Pancreatography demonstrated pancreatic body stenosis and extrapancreatic leakage of contrast agent at the stenosis site. Initially, the stenosis was partially dilated using a thin-tipped balloon catheter. Further caudal dilation was attempted; however, the catheter and another bougie dilator failed to pass through the stenosis. Subsequently, we dilated the stenosis using the SSR-7. When the SSR-7 was removed following dilatation, the tip was dislodged midway through the stenosis and remained in the pancreatic duct ( Video 1 ). An attempt to remove the tip using biopsy forceps failed because it could not pass through the stenosis. Subsequently, a wire-guided single-opening biopsy forceps (E634044, 2.2-mm channel; Olympus, Tokyo, Japan) was used to successfully grasp and remove the dislodged tip through the stenosis ( Fig. 2 ). The stenosis was dilated again using a thin-tipped balloon catheter, followed by the successful placement of a 5-Fr pancreatic stent at the stenosis site.

**Fig. 1:**
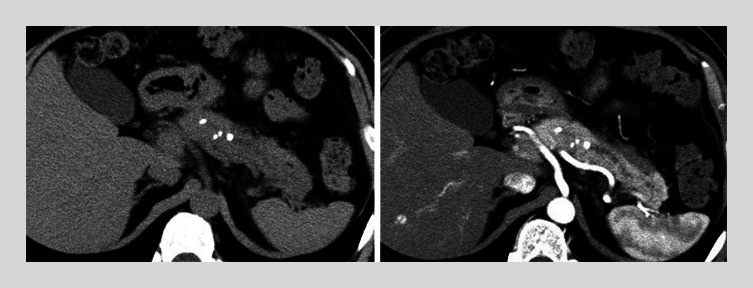
Abdominal computed tomography (CT) demonstrates pancreatic stones in the pancreatic body (left). The arterial phase of contrast-enhanced CT demonstrates pancreatic stones with dilation of the main pancreatic duct and inflammation around the pancreas (right).

**Fig. 2:**
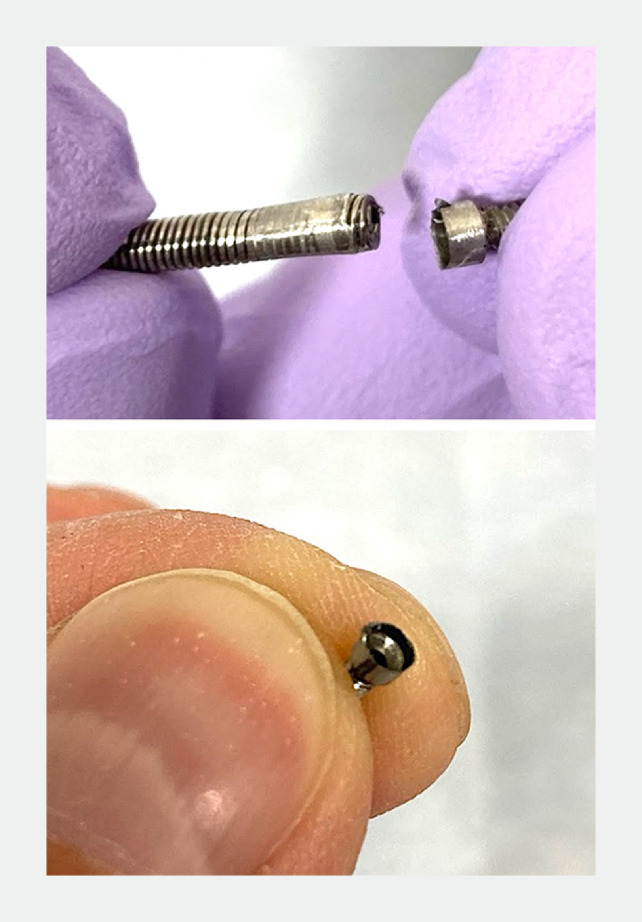
The Soehendra stent retriever tip is dislodged.

A wire-guided single-opening biopsy forceps successfully grasped the dislodged tip through the stenosis.Video 1

Endoscopy_UCTN_Code_CPL_1AK_2AZ

## Notes

Endoscopy E-Videos https://eref.thieme.de/e-videos
*E-Videos* is an open access online section of the journal *Endoscopy* , reporting on interesting cases and new techniques in gastroenterological endoscopy. All papers include a high-quality video and are published with a Creative Commons CC-BY license. Endoscopy E-Videos qualify for HINARI discounts and waivers and eligibility is automatically checked during the submission process. We grant 100% waivers to articles whose corresponding authors are based in Group A countries and 50% waivers to those who are based in Group B countries as classified by Research4Life (see: https://www.research4life.org/access/eligibility/ ). This section has its own submission website at https://mc.manuscriptcentral.com/e-videos .
